# Photothermally Antibacterial Piezoelectric Composite Dressing Synergized with Endogenous Electrical Stimulation for Wound Healing

**DOI:** 10.3390/pharmaceutics18050607

**Published:** 2026-05-15

**Authors:** Hao-Zhe Yu, Guan-Yong Deng, Nan Gao, Li-Hong Fan, Jian-Wen Wang, Xing-Jian Liu, Wei Zhang, Shi-Lin Tian, Yu-Xiong Weng, He-Shuang Dai, Yi-Wen Zhang, Huan Deng

**Affiliations:** 1 School of Chemistry, Chemical Engineering and Life Sciences, Wuhan University of Technology, Wuhan 430070, China; 346355@whut.edu.cn (H.-Z.Y.); 349065@whut.edu.cn (G.-Y.D.); lhfan@whut.edu.cn (L.-H.F.); 349070@whut.edu.cn (X.-J.L.); 346372@whut.edu.cn (W.Z.); 346344@whut.edu.cn (S.-L.T.); daiheshuang@whut.edu.cn (H.-S.D.); 374851@whut.edu.cn (Y.-W.Z.); 2 Department of Hand Surgery, Union Hospital, Tongji Medical College, Huazhong University of Science and Technology, Wuhan 430022, China; gaonan1112@gmail.com (N.G.); wjw19980315@163.com (J.-W.W.); yxweng1218@163.com (Y.-X.W.)

**Keywords:** conductive hydrogel, piezoelectric film, composite dressing, photothermal antibacterial, infected wound healing

## Abstract

**Background**: Photothermal therapy (PTT), a highly efficient and controllable method with minimal drug resistance, transforms near-infrared (NIR) radiation into heat. This process exerts antibacterial effects, aids in tissue repair, and promotes healing. **Methods**: Our study presented a novel kind of composite wound dressing that incorporated adhesive conductive hydrogel combined with piezoelectric film for NIR-responsive applications. The inherent adhesiveness of the hydrogel ensured robust anchoring of the piezoelectric film to both hydrogel matrix and wound site. Its conductivity enabled synergistic endogenous electrical stimulation with the piezoelectric film, while also serving as therapeutic layer to augment hemostasis, analgesia, and antibacterial activity. **Results**: The hydrogel’s capacity for moisture retention and exudate absorption sustained optimal wound environment, thereby supporting debridement and recovery. Furthermore, the piezoelectric film possessed excellent photothermal properties and transferred heat to the hydrogel through heat conduction to enhance antibacterial activity and promote wound healing. The in vitro and in vivo experiments confirmed that the composite dressing exhibited strong promotion effect on wound healing under NIR irradiation. **Conclusions**: In summary, our research provided a new strategy for developing advanced piezoelectric biomaterials with great clinical potential for wound healing.

## 1. Introduction

The skin, being the largest organ of the human body, serves as the primary line of defense. It is crucial in preserving homeostasis and facilitating normal physiological activities [1]. However, various incidents such as surgery, burns, abrasions, and cuts can result in trauma of varying degrees, some of which may necessitate long-term treatment [2,3]. Conventional wound care strategies predominantly involve the application of wound dressings, which provide protection, hemostasis, and absorption of wound exudates [4]. In contrast, novel wound dressings exhibit superior biocompatibility. For instance, hydrogel-based dressings can maintain a moist wound environment, absorb tissue exudates, and facilitate therapeutic agent delivery while stimulating the healing response [5]. However, they also have limitations such as poor mechanical properties and the inability to heal chronic wounds.

Existing studies have confirmed that electrical stimulation can accelerate wound healing by promoting cell proliferation and neovascularization during the wound healing stage [6,7,8]. Especially in the context of chronic wounds, electrical stimulation has been shown to mitigate pain perception through the activation of the nerve conduction system [9,10]. However, the current electrical stimulation devices for wound treatment in clinical practice have limitations such as inconvenient operation, poor portability and frequent charging. Therefore, alternative methods to obtain endogenous electrical energy must be considered [11,12,13,14]. Piezoelectric materials, characterized by their high piezoelectric properties, are explored to effectively address the aforementioned issues. These materials are capable of converting mechanical kinetic energy into endogenous electrical energy, thereby providing continuous internal electrical stimulation to accelerate wound healing. Poly(vinylidene fluoride-co-trifluoroethylene) (PVFT) is a kind of piezoelectric polymer material synthesized by the copolymerization of polyvinylidene fluoride (PVDF) with the functional, fluorine-containing monomer TrFE. This process significantly improves the dielectric coefficient, dielectric strength, and piezoelectric coefficient of PVFT [15]. Moreover, it also endows PVFT with enhanced crystallization, superior ferroelectric properties, and a more pronounced piezoelectric response, making it more suitable for most electromechanical conversion scenarios. However, both PVFT and most organic piezoelectric polymers are characterized by low output voltage and energy, while their surface hydrophobicity limits their application in biomedicine [16,17]. To further improve the piezoelectric properties of PVFT copolymers, a common strategy that introduces additional phases, such as lead zirconate titanate (PZT), barium titanate (BTO), graphene, metal electrode or zinc oxide (ZnO), is explored [18]. These additives can significantly enhance the electrical response of the polymer matrix [19]. Furthermore, MXene possesses abundant surface functional groups, high specific surface area, and exceptionally high electrical conductivity. These properties enable it to interact with the dipole of the PVFT molecular chain, thereby enhancing the piezoelectric performance. Zhao et al. [20] combined MXene and PVDF through electrospinning to produce piezoelectric hybrid membrane, which significantly enhanced the piezoelectric output and mechanical properties of PVDF. Concurrently, MXene serves as an effective photothermal agent. When exposed to near-infrared (NIR) irradiation, MXene nanosheets efficiently transform absorbed light energy into thermal energy. He et al. [21] developed a NIR-radiation-triggered photothermal–chemical synergistic treatment system based on DNA hydrogel by combining MXene nanosheets with DNA hydrogel for efficient local cancer treatment. Thus, utilizing MXene thoroughly mixed with the PVFT piezoelectric polymer could be served as electroactive material (P/M composite). Moreover, the P/M composite could be casted into film (P/M film) by tape casting method, which is expected to have excellent electrical properties.

However, the inadequate integration of a single-layer piezoelectric film with the wound can result in suboptimal bioelectric field transmission during wound repair. This compromises the efficacy of the piezoelectric material and fails to meet the complex environmental demands of the wound site [22]. Conductive hydrogels have been shown to promote fibroblast adhesion, proliferation, and migration, which are critical to wound healing [23]. Conductive polymers are frequently utilized in the fabrication of these hydrogels owing to their advantageous properties, including biocompatibility, oxidation resistance, and photothermal characteristics. Moreover, the electrical activity inherent to conductive polymers establishes an optimal physiological microenvironment that enhances cell migration, adhesion, and fibroblast proliferation, thereby accelerating the wound healing process.

Polyaniline (PANI) is readily synthesized and exists in various redox forms. The incorporation of PANI into hydrogels could enhance their elasticity and conductivity, thereby promoting the transport of nutrients, drugs, and metabolites [24]. Li et al. synthesized injectable conductive interpenetrating polymer network hydrogels with enhanced mechanical properties and good biocompatibility by adding PANI. The hydrogel showed the formation of an interpenetrated polymer network that meets the standards required for biomedical applications [25]. Conductive hydrogels based on PANI combine the advantages of hydrogels and organic conductive materials, which can significantly improve electrical conductivity and response to external stimuli while maintaining the inherent properties of the hydrogel [26]. Simultaneously, tannic acid (TA) possesses phenolic hydroxyl groups, which impart favorable skin affinity and robust adhesive capabilities to the hydrogel. The incorporation of Fe^3+^ into the TA-containing hydrogel has also been shown to significantly enhance its flexibility and adhesion properties [27].

**Scheme 1:**
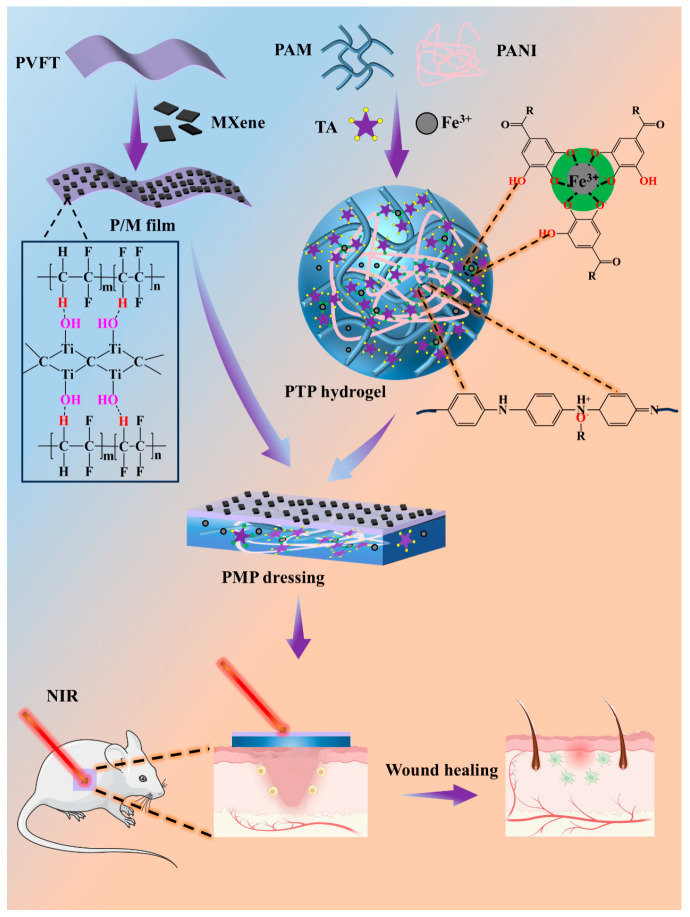
Schematic illustration of the preparation and wound healing property of the piezoelectric composite dressing.

Therefore, in our research, PTP conductive adhesive hydrogels were synthesized via one-pot method using acrylamide (AM), TA, and ANI as monomers. A novel kind of piezoelectric composite dressing with dual photothermal antibacterial functionalities and endogenous electrical stimulation was developed by integrating P/M composite film with PTP hydrogel (Scheme 1). The composite film and hydrogel were characterized using Fourier transform infrared spectroscopy (FT-IR), scanning electron microscopy (SEM), X-ray diffraction (XRD), and thermogravimetry and differential thermogravimetry (TG-DTG). In addition, the mechanical and electrical properties of MXene composite membranes and the swelling, mechanical, and adhesion properties of PANI hydrogels were evaluated at various concentrations to identify the most suitable materials for integration. The composite dressing was fabricated by combining piezoelectric film with conductive hydrogel and the piezoelectric, photothermal, and antibacterial properties of this composite material were evaluated. Subsequently, the therapeutic efficacy of the composite dressing on infected wounds was verified through in vivo animal experiments. This research provided a novel strategy for designing composite piezoelectric biomaterials for wound management and demonstrated their significant potential for clinical application.

## 2. Materials and Methods

### 2.1. Materials

Poly(vinylidene fluoride-trifluoroethylene) (PVFT, FC-30) was purchased from Acoma (Pierre-Bénite, France). Ti3C2TX MXene was purchased from BKNANAO Materials Co., Ltd. (Suzhou, China). N,N-dimethylformamide (DMF, AR), ferric chloride (FeCl3, AR), acrylamide (AM, CP), ammonium persulfate (APS, AR), tetramethylethylenediamine (TEMED, 99%), and aniline (ANI, AR) were purchased from Chemical Reagents Co., Ltd. (Nanjing, China). Tannic acid (TA, AR) and N,N-methylenebisacrylamide (MBA, AR) were purchased from McLean Biochemical Technology Co., Ltd. (Shanghai, China). *Escherichia coli* (*E. coli*, ATCC25922) and *Staphylococcus aureus* (*S. aureus*, CMCC (B) 26003) strains were purchased from the Biological Treasure Center (Beijing, China). Gibco MEM medium was obtained from Zhongqiao Xinzhou Biotechnology Co., Ltd. (Shanghai, China). The CCK-8 kit and Live/Dead Assay kit were purchased from Beyotime Biotechnology Co., Ltd. (Shanghai, China). All aqueous solutions were prepared using ultrapure water generated by MST-11-10 water purification system from Mosu Scientific Equipment Co., Ltd. (Shanghai, China).

### 2.2. Cell Lines and Animals

L929 cells were obtained from ATCC (NCTC clone 929-CCL-1). The cells were cultured in RPMI 1640 medium supplied with 10% *v*/*v* of FBS, 100 U/mL of penicillin, and 100 μg/mL of streptomycin. The cells were incubated in a 37 °C humidified environment with 5% CO_2_ supply. Six-week-old BALB/c mice (18–20 g, female) were obtained from Hubei Provincial Center for Disease Control and Prevention, Wuhan, China. All animal experiments were performed under the guidelines approved by the regulations of Chinese law and the local Ethical Committee. The animal experiments were approved by the Institutional Animal Care and Use Committee (IACUC Number: 4441).

### 2.3. Preparation of the P/M Composite Membrane

The P/M composite membrane was prepared by tape casting method. Firstly, 0.25 g of MXene was dissolved in 50 mL of DMF solution, stirred at room temperature for 30 min and then sonicated for 10 min to obtain a uniformly dispersed 5 mg/mL MXene dispersion. Then, 1 g PVFT powder was added into DMF, stirred at room temperature (RT) for 3 h, and then ultrasonically treated to obtain mixed PVFT solution. Then, MXene dispersion was added, and the ultrasonic stirring was continued to make the solution uniform. The solution was then dumped on a clean glass plate and placed in a vacuum drying oven for 12 h at a temperature of 50 °C. Finally, the temperature of the vacuum drying oven was adjusted to 120 °C for 2 h, and then the glass plate was immersed in deionized water. The film was washed with deionized water after falling off from the surface of the glass plate, and then dried.

The specific parameters were shown in Table 1 below. The film was washed with deionized water after falling off from the surface of the glass plate, and then dried. The prepared composite film was cut into 60 × 60 mm^2^ sample specifications for piezoelectric polarization. The specific parameters were set as follows: the polarization voltage was 24 kV, the polarization time was 30 min, the distance between the sample and the polarization needle was set to 20 cm, and the polarization was carried out under dry conditions at RT (the P/M composite membrane was the P/M-1.00 composite membrane by default).

### 2.4. Fabrication of PTP Conductive Hydrogels

The preparation of PTP hydrogel was completed by one-pot method. A certain amount of AM was uniformly dissolved in ionic water (32 wt %, 7.6 mL), and then 0.27 g of TA, 0.1 g of ferric chloride and 0.2 g of MBA were dissolved in 10 mL of deionized water. Then, a certain amount of TA, Fe^3+^, MBA solution and aniline (ANI) were added to the AM solution to continue stirring, and then an appropriate amount of TEMED and APS was immediately added. Then, the PTP hydrogel was obtained by uniformly mixing and standing for a period of time and placing it in an oven at 60 °C. Each was named according to different ANI content; for example, ANI with the content of 0.02 mL was named PTP-0.02 (PTP hydrogel defaults to PTP-0.06).

### 2.5. Characterization of P/M Composite Membrane

FT-IR can be used to study the chemical structure and composition of the samples. The specific operation steps were as follows: the P/M composite film was cut into a suitable shape and placed in the drying box for 30 min to remove moisture. Then, the film was pressed with a clamp and vacuumized for scanning, with the scanning range of 4000–400 cm^−1^.

The crystal structure of P/M composite membrane was detected by XRD. The P/M composite film was cut into a square of 20 × 20 mm^2^, and the crystal phase was analyzed by X-ray diffractometer. The target was Cu-Kα target, the scanning speed was 2°/s, and the detection range was 10–80°.

The surface morphology of the P/M composite membrane was observed. Firstly, the prepared composite membrane was washed with ethanol–water solution, and then rinsed repeatedly with deionized water and dried. The P/M composite film was cut into a suitable shape and adhered to the test bench. Then, vacuum treatment and surface gold spraying were carried out to observe the surface morphology and obtain pictures.

### 2.6. Characterization of Hydrogels

The functional groups of the prepared hydrogel samples were characterized by Fourier transform infrared spectroscopy. The freeze-dried hydrogel samples were ground into powder and dried at 50 °C for 6 h in a blast drying oven. The samples were mixed with potassium bromide (KBr) powder in a certain proportion, ground and pressed, and then tested. The scanning test range was 400–4000 cm^−1^.

The crystal structure and properties of the samples were analyzed by X-ray diffractometer. The processed freeze-dried hydrogel sample powder was placed in the sample scaffold, and the test diffraction angle range was set to 5–90°.

Each hydrogel sample was frozen and crisped in liquid nitrogen, and then freeze- dried for 48 h. The sample was fixed on the sample holder using conductive adhesive, and then gold was sprayed on the cross-section of the sample. The internal microstructure of the hydrogel was observed under a scanning electron microscope.

The hydrogel sample after freeze-drying treatment was cut into very fine particles, and then the vacuum drying oven was used to remove water. Then, a certain amount of sample was weighed, put into the instrument and connected to N_2_, and the weight change of the sample from RT to 1000 °C was tested.

### 2.7. Water Contact Angle Assay

The hydrophilicity of P/M composite piezoelectric film was evaluated by static contact angle test at RT. The specific test steps were as follows: the knob to spin a droplet of the appropriate size at the tip of the needle was controlled, and then the height of the plane where the composite film was located was increased until the droplet dropped on the surface of the film. After the shape of the water droplets was stable, a clear image was taken, and then the image data was processed to calculate the contact angle of the composite film. In order to ensure the authenticity and reliability of the data, each group of composite membranes was subjected to three parallel tests, and the results were averaged.

### 2.8. Swelling Performance of PTP Conductive Hydrogels

In order to evaluate the water absorption capacity and swelling stability of PTP hydrogels, the swelling ratio needed to be tested. The hydrogel samples were prepared to ensure the uniform shape and size of the samples. The hydrogel samples were freeze-dried for 48 h to constant weight (Mt). The freeze-dried hydrogels were immersed in PBS buffer at pH 7.4 at 37 °C. During the soaking process, the hydrogel sample fully absorbed water and reached an equilibrium state. At regular intervals, the soaked hydrogel samples were taken out and the excess water on the surface was gently sucked with filter paper, and the weight was weighed (recorded as M_0_). Continuous measurements were taken until the hydrogel reached stable swelling equilibrium state. In order to ensure the accuracy of the data, each group of hydrogel samples were tested three times in parallel. The swelling ratio of the hydrogel was calculated according to Formula (1) [28,29]:

(1) $$Swelling\;ratio=\frac{M_{t}-M_{0}}{M_{0}}\times 100\%$$

### 2.9. Mechanical Properties

The composite piezoelectric film was cut into a rectangular size of 60 × 10 mm^2^, and the upper and lower ends of the film were pasted with cotton tape to facilitate fixing and increasing the friction force. The thickness and width of the composite film were measured using vernier caliper. At RT, the tensile test of the composite film was carried out at the speed of 8 mm/min by using an electronic universal material testing machine. According to the measured stress–strain data curve, three parallel tests were performed on each film using the same method, and the results were averaged.

The hydrogel was prepared into a dumbbell shape with a size of 20 mm long, 3 mm wide and 1 mm thick. The electronic universal material testing machine was used to test the hydrogel at the speed of 5 mm/min, and the stress–strain data curve was measured. The hydrogel was subjected to three parallel tests using the same method, and the results were averaged.

The hydrogel was prepared into a cylinder with a diameter of 25 mm and a height of 20 mm, and equilibrated for 6 h after gelation. The hydrogel sample was placed on the machine to apply the compression force, and the sample was compressed at a speed of 20 mm/min at RT with the universal testing machine. The instrument was compressed to the critical point of the hydrogel sample to obtain the stress–strain curve of the hydrogel sample. In order to ensure the accuracy of the data, each group of hydrogel samples was tested three times in parallel.

Rheological measurements were performed on a rheometer (DHR-2, TA Instruments (New Castle, DE, USA)) to prepare disc-like hydrogel samples (20 mm × 2 mm). The gel–sol transition point of the hydrogel was determined by strain scanning in the strain range of 0.1–400% at a constant frequency of 1 Hz at 37 °C. Under the condition of constant strain amplitude γ = 5%, the frequency sweep was carried out in the frequency range of 0.1~10 Hz.

### 2.10. Tissue Adhesion of Hydrogels

The adhesion ability of the hydrogel was tested by the lap shear method, and the pig skin was used to simulate the human surface to test the adhesion of the hydrogel. Firstly, the fresh pig skin was treated cleanly, cut into a long strip shape of 40 × 10 mm^2^, and the cut pig skin was soaked in PBS solution for 2 h. The prepared hydrogel was adhered between two pig skins. The contact area between the pig skin and the hydrogel was 10 × 10 mm^2^; then, standing for 1 h ensured that the hydrogel was fully adhered to the pig skin. Subsequently, the clamp was clamped and the tensile test was carried out using a universal material testing machine. When the two pig skins were separated, it had the maximum fracture strength. The magnitude of the force at this time was recorded and calculated according to the following formula. Each group of hydrogels was tested three times, and the experimental results were averaged. The hydrogel adhesion to pig skin and P/M film were also tested according to the above steps, and pig skin was replaced with composite film. The bonding strength is obtained from Formula (2):

(2) $$Adhesive\;strength\left(\mathrm{kPa}\right)=\frac{F}{S},$$

Among them, F was the maximum fracture force, S was the contact area (cm^2^) between the hydrogel and the pig skin.

### 2.11. Degradation Performance Test of Hydrogels

The freeze-dried hydrogel samples were weighed and recorded as G_A_, and all immersed in 10 mL of simulated body fluid, and then placed in a shaker, 160 rpm, 37 °C. These samples were taken from simulated body fluids at different time points (1, 4, 7, 10 and 14 d) and freeze-dried, and their weights were recorded as G_B_. In order to ensure accuracy, each group was averaged after three parallel tests. The degradation rate of the hydrogel was calculated according to the Formula (3):

(3) $$Degradation\;rate=\frac{G_{A}-G_{B}}{G_{A}}\times 100\%$$

### 2.12. Electrical Conductivity of PTP Conductive Hydrogels

In order to explore the effect of adding PANI on the conductivity of hydrogels, the resistance of hydrogels was tested by electrochemical workstation. The prepared hydrogel was cut into a cuboid of appropriate size, and the resistance of the hydrogel was measured by an electrochemical workstation with electrodes connected at both ends. The conductivity σ of the hydrogel was calculated according to Formula (4):

(4) $$\sigma =\frac{L}{R\times S},$$

L was the length of the test hydrogel, R and S represented the resistance and cross-sectional area of the hydrogel sample, respectively.

### 2.13. Electrical Properties of Piezoelectric P/M Film

In the piezoelectric coefficient test, the P/M composite film was first smoothed to ensure that the surface had no obvious defects. The sensor was connected to the surface of the sample. The instrument automatically processed the piezoelectric coefficient d_33_ according to the pressure applied to the sample surface and the corresponding charge signal. The same method was used to test three positions randomly selected for each membrane, and the results were averaged.

The ferroelectric performance test ensured that the surface of the P/M composite film was flat and defect-free, and cut into an appropriate size. The electrode was coated on the surface of the sample to ensure that the electrode was in good contact with the surface of the sample and the area size of the electrode was appropriate. The sample was placed in a test device and an applied electric field was applied with an electric field intensity of 40 kV/mm and a frequency of 25 Hz. Then, measurement results were recorded. For the accuracy of the data, each group of composite membranes was tested in parallel three times, and the results were averaged.

For the open circuit voltage and conductive current test, the P/M composite film was cut to suitable size, and its front and back sides were tightly connected with the conductive copper foil, respectively. The surface of the copper foil was covered with a layer of PET plastic to protect the internal device. Then, the copper foil was completely in contact with the measuring electrode of the electrochemical workstation. A mechanical arm was used to apply pressure perpendicular to the membrane surface to ensure that the force applied was consistent in size and frequency. The open circuit voltage measured by the measuring instrument was recorded. Each group of composite membranes was tested three times in parallel, and the results were averaged. The P/M composite film was cut to suitable size, and the prepared hydrogel was cut into a shape of suitable size. The piezoelectric film was bonded with the hydrogel to form composite dressing. A mechanical arm was used to apply pressure perpendicular to the membrane surface to ensure that the force applied was consistent in size and frequency. The open circuit voltage measured by the measuring instrument was recorded. Each group of composite membranes was tested three times in parallel, and the results were averaged.

For the surface potential test and surface potential stability test, surface potential was an important method to evaluate the piezoelectric properties and surface charge distribution of composite films. The P/M composite film was cut to appropriate size, and the surface potential measuring instrument was connected to the electrode of the sample to measure the potential distribution on the surface of the sample. It was ensured that the grounding connection of the measuring instrument was good to avoid interference and error. Each group of composite membranes was tested in parallel three times, and the results were averaged. In order to explore the stability of the surface potential of the P/M composite membrane, the composite membrane was placed in Eagle medium for 28 days. After the composite membrane sample was taken out every 1 week to rinse its surface, surface potential test was performed, and the above steps were repeated for testing.

### 2.14. Photothermal Performance Evaluation

The 808 nm near-infrared laser was used to irradiate the piezoelectric film, hydrogel and composite dressing at a distance of 15 cm from the near-infrared laser, and the power was 1.0 W/cm^2^. Three cycles of 5 s, 15 s and 30 s were used to irradiate the composite dressing. The irradiation time of the hydrogel sample was 10 min, and the PTP hydrogel was also irradiated for 30 s as a control of the composite dressing. A near-infrared thermal imager was used to record the temperature change and take pictures.

### 2.15. Antibacterial Assay In Vitro

The antibacterial activity of hydrogels and composite dressings against *Staphylococcus aureus* and *Escherichia coli* was tested and evaluated. After the two strains were activated, they were cultured for 24 h. A certain amount of the prepared hydrogel was added to the 24-well culture plate, and then moved into the ultra-clean bench for ultraviolet sterilization. A 100 μL bacterial suspension (PBS, 10^6^ CFU/mL) was added to the surface of the hydrogel disc, and then the 24-well culture plate was cultured at 37 °C for 2 h. The hydrogel group was irradiated with an 808 nm NIR laser for different times (3 min, 5 min, 10 min) and without irradiation. The composite dressing group was irradiated with the 808 nm NIR laser for 30 s cycles (2, 3, 5 cycles), and continued to be cultured at 37 °C for 2 h. PBS was added to each well to re-suspend the bacteria. As a negative control group, 100 μL sterile PBS (10^6^ CFU/mL) bacterial suspension was added to 1 mL PBS. Then, 50 μL was evenly applied to the solid agar plate. After incubation at 37 °C for 18–24 h, the number of colonies on the plate was observed and recorded. In order to ensure the accuracy of the data, each group of samples were tested at least three times, and the results were averaged. The antibacterial Formula (5) is as follows:

(5) $$Antibacterial\;ratio=\frac{M_{0}-M_{1}}{M_{0}}\times 100\%$$

Among them, M_0_ and M_1_ represent the average number of colonies on blank plates and sample plates, respectively.

### 2.16. In Vitro Biocompatibility and Cell Scratch Assay

L929 cells were cultured in MEM medium supplemented with 10% fetal bovine serum and 1% penicillin–streptomycin. The culture was maintained in a controlled environment of 37 °C, 5% CO_2_ and humidified air, and the medium was changed every other day. L929 cells were seeded in 96-well plates and co-cultured with various biomaterials. A CCK-8 kit was used for viability assessment. The evaluation procedure consisted of dispensing the CCK-8 solution into each well and incubating the plate at 37 °C for 2 h. The absorbance was then measured at 450 nm using the Varioskan TM LUX Microplate Reader (Thermo Fisher Scientific Inc., Waltham, MA, USA) to determine the number of living cells based on the OD450 value. The cytotoxicity was evaluated 3 days after seeding. The experimental protocol included the staining of living cells with calcein-AM and non-living cells with propidium iodide. The results were observed with a fluorescence microscope.

The blood obtained from SD rats was diluted to obtain 2% red blood cell (RBC) suspension. The material was ground into powder, 25 mg was weighed and placed in a test tube, and 10 mL normal saline was slowly dripped into the test tube. After it was completely dissolved/dispersed, the test tube was placed in a water bath for 0.5 h at 37 °C. The diluted anticoagulant whole blood 0.1 mL was taken to the test tube, shaken well, placed in a water bath, and continued for 1 h at 37 °C. The test tube was taken out, and the blood cells were centrifuged at a speed of 1500 rpm/min for 10 min. The supernatant was taken out and placed in an ultraviolet spectrophotometer for absorbance determination. The wavelength was set to 545 nm. The hemolysis rate (HR) of the sample was calculated according to Formula (6):

(6) $$HR=\frac{A_{1}-A_{3}}{A_{2}-A_{3}}\times 100\%$$

In the equation, HR meant hemolysis rate, the absorbance of the A_1_ was sample group, the absorbance of the A_2_ was the positive control group (10 mL deionized water, 0.1 mL anticoagulant whole blood, no sample material), and the absorbance of the A_3_ was the negative control group (10 mL normal saline, 0.1 mL anticoagulant whole blood, no sample material). The experiment was repeated five times.

The migration of L929 cells was studied by a special wound model of culture insertion in two wells (ibidi GmbH, Martinsried, Germany). Approximately 70 mL (4 × 10^5^ cells/mL) of the cell suspension was placed into a hole in a culture plate on a 12-well plate. After incubation in complete medium for 24 h, the cells converged around the insert, forming a 500 mm wide gap when the insert was removed. Then, the cells were co-cultured with PT hydrogel extract (1 mg/mL) and PTP hydrogel extract for 12, 24 or 36 h, respectively. In the P/M group, the positive and negative sides of the membrane were closely connected with the conductive copper foil, respectively, and inserted into the wire. The surface of the copper foil was covered with a layer of PET plastic to protect the internal device. The wire was placed in the cell culture medium, and then the force generation device was knocked to make the membrane produce electrical stimulation (knocking for 15 min every 12 h). In the PMP group, P/M was replaced with PMP, and PTP hydrogel extract was added to the cell culture medium. Cell migration was continuously monitored and quantitatively evaluated at each interval using an inverted microscope and ImageJ software. The cell migration rate was as follows:

(7) $$Cellmigrationrate=\frac{A_{0}-A_{t}}{A_{0}}\times 100\%$$

A_0_ and A_t_ were the initial scratch area and the scratch area after a period of culture, respectively.

### 2.17. In Vivo Wound Healing Analysis

All animal experiments were approved by the Animal Protection Committee of Tongji Medical College. Six-week-old female BALB/c mice were anesthetized, depilated and disinfected on the back, and then underwent full-thickness wound resection with a diameter of 10 mm. Each wound was injected with 10 μL (1 × 10^7^ CFU/mL) *Staphylococcus aureus* suspension. The wound was then covered with a film dressing for 2 days to simulate the infected wound surface. The mice were divided into five experimental groups: control group, medical dressing water glue group, PTP hydrogel+NIR group (808 nm, 1 W/cm^2^ for 10 min), PMP, PMP+NIR group (808 nm, 1 W/cm^2^ for five cycles) (n = 6). In all composite dressing groups, the piezoelectric effect was generated through deformation of the composite membrane induced by the natural movements and joint bending of the mice.

Wound tissues were collected on day 2, 5, 8 and 11 after injury. Wound tissues were collected on day 14, fixed with 4% paraformaldehyde, stained with hematoxylin and eosin (H&E), and stained with Masson’s trichrome to evaluate granulation tissue width and collagen deposition, respectively. Fiji 2.14.0/1.54f (ImageJ Foundation, Madison, WI, USA) was used to collect wound images and analyze them. The relative wound area calculation Formula (8) is as follows:

(8) $$Relative\;wound\;area\;=\;\left(\frac{{Area}_{n}}{{Area}_{0}}\right)\times \;100\%$$

Area_0_ was the initial wound area, and Area_n_ was the wound area on the 2nd, 5th, 8th and 11th day, respectively.

### 2.18. Statistical Analysis

All numerical data were presented as mean ± standard deviation (SD) and statistically analyzed using GraphPad Prism 9.0 with three or more replicate values. Significant differences were determined using Student’s *t*-test for comparison of two groups and one-way ANOVA with post hoc Tukey’s test for multiple comparisons. The statistical significance was declared when * *p* < 0.05, ** *p* < 0.01, *** *p* < 0.001 and **** *p* < 0.0001.

## 3. Results and Discussions

### 3.1. Preparation and Characterization of P/M Composite

**Figure 1:**
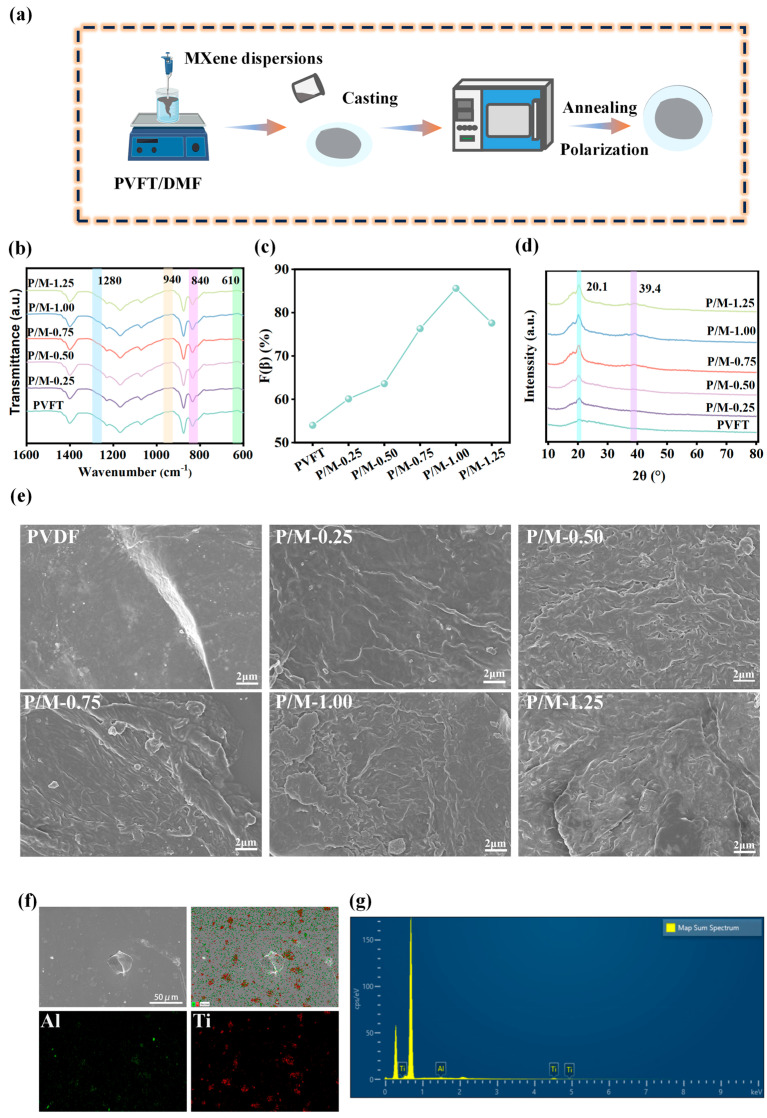
(**a**) Preparation process of P/M composite film. (**b**) FTIR, (**c**) β phase content, and (**d**) XRD spectra of composite membranes with different MXene contents. (**e**) SEM images of composite membranes with different MXene concentrations. Scale: 2 μm. (**f**) SEM-EDS images and (**g**) EDS energy spectra of P/M-1.00 composite membranes. Scale: 50 μm.

The preparation process of the P/M composite membrane was shown in Figure 1a, and the appearance of the successfully prepared P/M composite film was shown in Figure S1a. It can be seen from the figures that the appearance of the composite film was transparent and black, with smooth surface and uniform thickness, and the side view showed that the thickness was thinner. The uniformly grayish-black coloration of the film, without any discernible color variation, suggested homogeneous dispersion of MXene throughout the material. Figure S1b illustrated the two-step preparation mechanism for the P/M composite film: casting and subsequent polarization. PVFT exhibited five primary crystalline phases (α, β, γ, δ, and ε), with its piezoelectric properties originating predominantly from the β phase. This was attributed to the β phase possessing the highest spontaneous polarization intensity; consequently, an increased proportion of this phase correlated with enhanced piezoelectric performance in PVFT [30]. In this study, MXene was thoroughly mixed with PVFT/DMF solution to fabricate a cast film. The functional groups on the surface of the MXene nanosheets were brought into full contact with PVFT molecules, interacting at the interface to form hydrogen bonds that effectively lock and anchor dipoles. Subsequent polarization under high-voltage electric field promoted the optimal alignment of these dipoles and the formation of β phase with high piezoelectric properties. Furthermore, the excellent electrical conductivity of MXene was also beneficial for enhancing the voltage output.

According to Figure 1b, the characteristic vibration bands of α crystalline phase in FTIR were at 610 and 940 cm^−1^ and that of polar β crystalline phase was clearly observed at 1280 and 840 cm^−1^. The characteristic peak at 840 cm^−1^ corresponded to asymmetric stretching vibrations of –CF2. Based on the characteristic vibration bands at 610 and 840 cm^−1^, the content of β crystalline phase in composite films was calculated using Formula (9):

(9) $$\;F(\beta )=\frac{A_{\beta }}{1.26A_{\alpha }+A_{\beta }}\times 100\%$$

Among them, A_α_ and A_β_ represented the vibration bands at 690 cm^−1^ and 840 cm^−1^, respectively.

The β crystalline phase proportion of the composite films increased from 54% for pristine PVFT to 85.6% for 1.00% MXene and then decreased to 77.6% for 1.25% MXene (Figure 1c). To further reveal the contribution of β to electrical properties, XRD tests were performed on PVFT composite films with different MXene contents. The results were shown in Figure 1d. The characteristic peaks at 2θ = 20.1° and 39.4° ((110) and (001) crystallographic planes) correspond to the β and α crystalline phases, respectively. The results showed that as the MXene content increased, the intensity of the characteristic peak of the β crystalline phase (2θ = 20.2°) increased and enhanced, reaching a maximum at 1.00% MXene. After exceeding this content, the peak intensity was slightly weakened. This was because the surface functional groups of MXene and PVFT can interact with each other, which facilitated the formation of F–H and O–H hydrogen bonds and uniform dispersion of MXene in the PVFT membrane. When the MXene content was too high, the MXene nanosheets began to agglomerate. Based on the data results, it was judged that the composite film with 1.00% MXene content might have the best electrical properties.

The surface morphology of films with different contents at the same magnification was shown in Figure 1e. It can be seen that the film surface of PVFT was relatively smooth and had no protrusions. This indicated that PVFT molecules were closely arranged to form dense surface. When the content of MXene was 0.25% and 0.50%, obvious chain structures began to appear on the film surface, which proved that the β crystal phase content in PVFT began to increase. When the MXene content increased from 0.75% to 1.25%, protrusions and obvious stratification began to appear on the membrane. Especially when the MXene content was 1.25%, the number of chain structures representing the β crystal phase began to decrease. This may be because when the MXene content was too much, agglomeration occurred, resulting in uneven distribution and reducing the combination of abundant functional groups on the MXene surface with PVFT, which may have affected the electrical properties of the composite film. The Ti and Al elements of P/M-1.00 composite film were evenly distributed on the surface of the film, which indicated that the MXene nanosheets were uniformly distributed on the PVFT film (Figure 1f). Figure 1g showed the type and proportion of each element on the P/M-1.00 composite film, and also showed that the MXene nanosheets were uniformly distributed on the PVFT film.

### 3.2. Synthesis and Characterization of PTP Conductive Hydrogels

**Figure 2:**
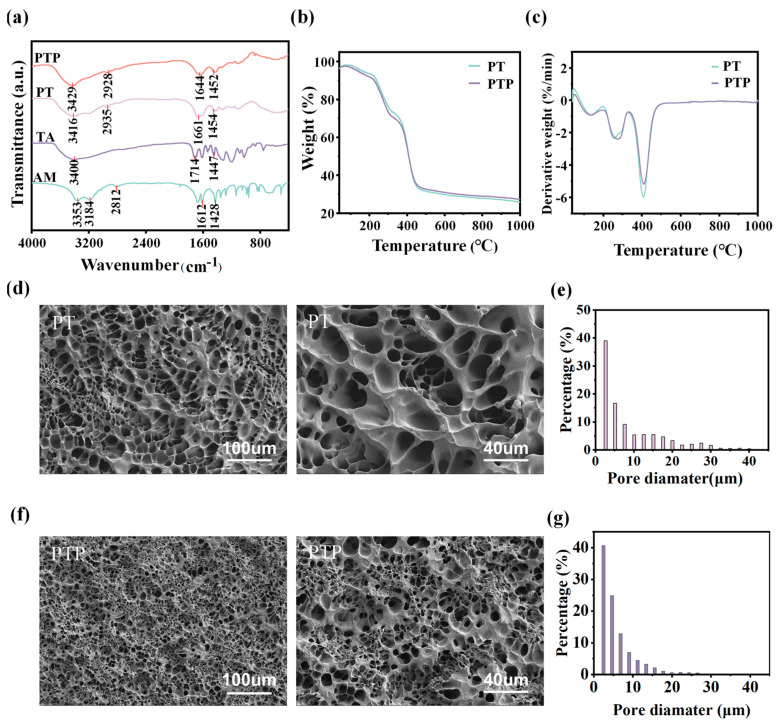
(**a**) FTIR spectra of AM, TA, PT hydrogel and PTP hydrogel. (**b**) TG and (**c**) DTG profiles of PT and PTP hydrogel. (**d**) SEM images of PT hydrogels at different magnifications. Scale: 100 μm and 40 μm. (**e**) Pore size distribution of PT hydrogel. (**f**) SEM images of PTP hydrogels at different magnifications. Scale: 100 μm and 40 μm. (**g**) Pore size distribution of PTP hydrogel.

The PTP hydrogel was synthesized using the one-pot method. The FT-IR spectra of the PT and PTP hydrogels are presented in Figure 2a. In the infrared spectrum of AM, the peak at 1612 cm^−1^ corresponded to the bending vibration of the amino (N-H) group, while the absorption peaks at 3184 cm^−1^ and 3353 cm^−1^ represented the stretching vibrations of the same group. The stretching vibration peaks for the cyano (-CN) and methylene (-CH2) groups appeared at 1428 cm^−1^ and 2812 cm^−1^, respectively. For TA, the spectrum revealed an absorption peak at 3400 cm^−1^, indicating the presence of hydroxyl (-OH) groups. Peaks at 1447 cm^−1^ and 1714 cm^−1^ corresponded to the stretching vibration of the carbon chain in the aromatic hydrocarbon and the carbonyl (-C=O) group, respectively, which was consistent with previous research findings [31]. The FT-IR spectrum of the PTP hydrogel revealed characteristic peaks attributable to (C-N) tensile vibrations and benzene ring stretching, observed at 1281 cm^−1^ and 1452 cm^−1^, respectively [32]. The spectra of the PTP hydrogel exhibited shift relative to those of PT, TA, and AM, indicating the formation of hydrogen bonds among PAM, TA, and PANI. This interaction caused the stretching vibration peak to shift toward the high-frequency region. A similar phenomenon was also observed in the FT-IR spectra of the PTP hydrogel compared with that of AM, which confirmed the successful synthesis of the PTP hydrogel. The thermal stability of the hydrogel can provide information regarding its crosslinking density and component ratio. The XRD patterns of the PT and PTP hydrogels were presented in Figure S2. The XRD pattern of the PTP hydrogel revealed a relatively flat diffraction peak at 23.2°. Compared to the corresponding peak of the PT hydrogel, this peak exhibited increased intensity and sharpness. Furthermore, the XRD spectrum did not show a diffraction peak for PANI, indicating that it possessed an amorphous structure within the hydrogel. The increased peak intensity and sharpness suggest that the incorporation of PANI enhanced the crystallinity of the hydrogel, which can be attributed to the ordered arrangement and interaction of the PANI chains within the hydrogel network.

It can be seen from Figure 2b that the hydrogels have three stages of weight loss. The first stage of weight loss was due to the evaporation of adsorbed water and bound water, and the temperature ranges for the two hydrogels were 40–217 °C and 40–222 °C, respectively. This stage was also known as the “weight-loss stage”. The second stage was the main mass loss, at which point the intermolecular hydrogen bonds in the hydrogel began to break, and thermal decomposition and structural disintegration occurred. The temperature ranges were 217–446 °C and 222–452 °C, respectively. The third stage was the mass stabilization stage, where the main thermal decomposition ended, the rate of mass loss slowed down, and the curve tended toward stable state. The relative masses of the final residues were 25.82% and 27.22%, respectively. Figure 2c showed the DTG curves of PT and PTP hydrogels, indicating that the rate of decomposition of the hydrogels changes with increasing temperature. The sharp peak indicated rapid and precipitous thermal decomposition process for the hydrogel, whereas the gentler peak and curve suggested slower process. Three peaks were observed at identical positions in both the first and second stages for the two hydrogels. The maximum decomposition rate for the PT hydrogel occurred at 406.1 °C, while that of the PTP hydrogel was at 410.2 °C. Both hydrogels exhibited their maximum decomposition rate during the second stage. The addition of PANI reduced the maximum decomposition rate and increased the final residue mass, indicating that its introduction formed more hydrogen bonds and polymer carbon chains within the hydrogel, thereby improving its thermal stability.

The morphology of the prepared PT and PTP hydrogel samples was observed by SEM, and SEM images of both hydrogels were captured at two different magnifications. The corresponding reference scales are 100 μm and 40 μm, respectively (Figure 2d). The micrograph revealed that the PT hydrogel possessed continuous network structure with well-dispersed macroporous architecture. However, the distribution was not uniform, and smaller pores were also present. This phenomenon may be attributed to the complexation reaction between TA and Fe^3+^ and the formation of hydrogen bonds with PAM, which resulted in the coexistence of large pore networks and small pore regions within the PAM matrix. In contrast, the SEM images of the PTP hydrogel revealed a greater number of smaller pores (Figure 2f), indicating that the PANI network was successfully interspersed throughout the PAM network, thereby creating much denser porous structure. To investigate the effect of PANI incorporation on porosity, the pore size distribution of the SEM images for both hydrogels was statistically analyzed at the 100 μm scale (Figure 2e,g). The analysis yielded 858 and 2044 pores for the PT and PTP hydrogels, respectively. As shown in the figure, the proportion of pores in the 0–10 μm range was 64.8% for the PT hydrogel and 85.4% for the PTP hydrogel. These findings suggested that the addition of PANI created denser crosslinking network, which contributed to enhanced mechanical properties.

### 3.3. Water Contact Angle Test and Mechanical Properties of Composite Membrane

The surface water contact angle changes of the composite membranes with different MXene contents were shown in Figure S3. The water contact angle of the pure PVFT film was 88.9° ± 0.7°, which was close to the balance of hydrophilicity and hydrophobicity. With the increase of MXene content, the hydrophilicity of the material gradually increased, from 88.9° ± 0.7° of the PVFT membrane to 61.6° ± 1.7° of the P/M-1.25 composite membrane. This was due to the rich functional groups (such as-OH, -O, -F, etc.) on the surface of MXene nanosheets, which easily form hydrogen bonds with water. Adding MXene to the PVFT membrane can effectively improve its hydrophilicity.

**Figure 3:**
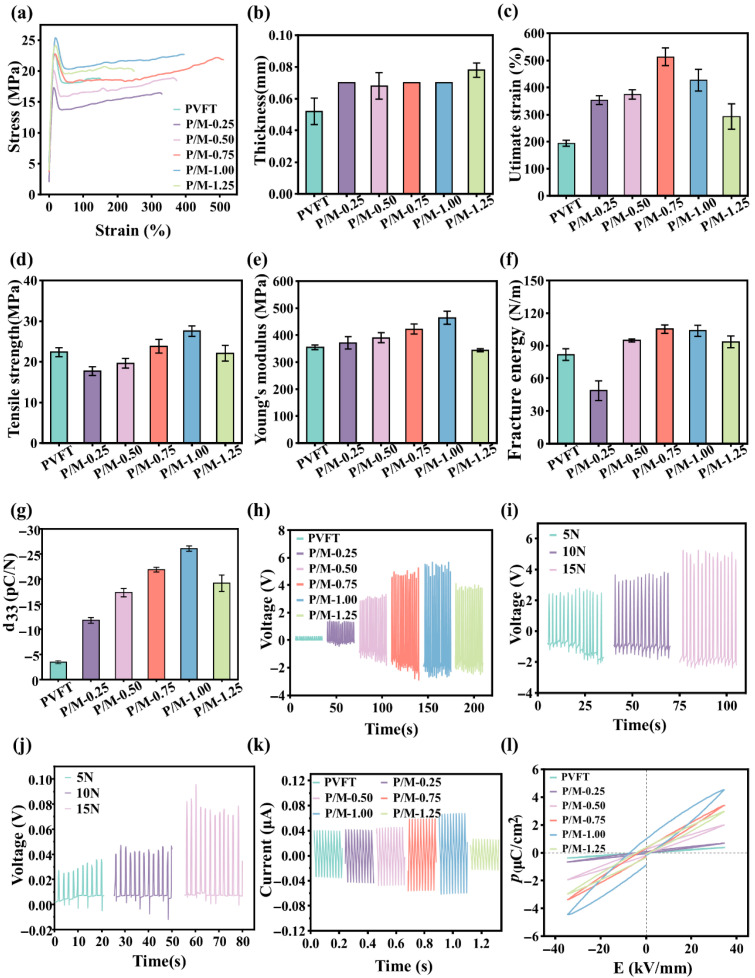
(**a**) Stress–strain curves, (**b**) film thickness, (**c**) maximum elongation at break, (**d**) tensile strength, (**e**) Young’s modulus, and (**f**) fracture energy of composite membranes with different concentrations of P/M (n = 3). (**g**) The d_33_ piezoelectric coefficients of P/M composite membranes at different concentrations (n = 3). (**h**) Piezoelectric output plots of P/M composite membranes with different contents at 15 N stress, (**i**) piezoelectric output of P/M-1.00 composite membranes under 5 N, 10 N and 15 N stresses. (**j**) Piezoelectric output of composite dressing under 5 N, 10 N and 15 N stresses. (**k**) Electrical conductivity and (**l**) P-E return line of P/M composite film with different contents.

When the P/M composite membrane acted on the wound, it not only needed to supply power to the wound through triboelectric and piezoelectric effects, but also played a supporting and protective role. The strength of mechanical properties will also affect the stability and durability of biomaterials. If the strength of the material was insufficient, it may lead to failure or deformation during the treatment process, thus affecting the therapeutic effect of the wound. Therefore, biomaterials should have good mechanical properties. The mechanical properties of the film were tested, and the two ends of the film were fixed on an electronic universal material testing machine for tensile testing. As shown in Figure 3a, all films exhibited elastic and plastic stages. Due to the influence of the plasticization induced by the phase transition between PVFT and MXene, the crystal structure of the film changed, making it have higher ductility and toughness. It can be observed from Figure 3b–d that the thickness of the PVFT film was between 0.04–0.06 mm, the upper yield point was 22.5 MPa, and the maximum elongation at break was 193.6%. This was due to the low viscosity of the solution when MXene was not added, and the film thickness poured by the casting method was thinner, which reached a critical point easily and quickly during the tensile process in the plastic stage (material is not uniform or defective). As a result, the deformation increased significantly, the effective tensile cross-section decreased sharply, the necking phenomenon occurred, and finally it was pulled off at the necking. Therefore, the thickness and maximum elongation at break of PVFT film were the smallest. When theMXene content increased, the viscosity of the solution increased, resulting in an increase in the film thickness from a minimum of 0.04 mm to a maximum of 0.08 mm. The maximum elongation at break first increased to 513% and then decreased to 292.9%, which was due to the increase of film thickness caused by the increase of MXene content in the early stage, which enhanced the ability of the film to resist deformation. With the continuous increase of MXene content, the content of β phase increased, resulting in a decrease in the proportion of α phase. Among the different crystalline phases of PVFT, the α crystalline phase exhibited the best mechanical properties, while the mechanical properties of the β phase were relatively poor. From Figure 3d–f, it can be observed that with the increase of MXene content, the tensile strength, Young’s modulus and fracture energy of the composite film began to show an increasing trend, and the mechanical properties decreased at 1.25% MXene. This may also be due to the agglomeration of excessive MXene, which reduced the uniformity of the composite film and led to stress concentration. Although the agglomeration of crystal phase and MXene affected the mechanical properties of the composite films, the maximum elongation at break of all composite films was more than 290%, the tensile strength was more than 15 MPa, and the Young’s modulus was 340 MPa. This was sufficient to meet the mechanical conditions required as a wound repair material.

### 3.4. Electrical Property of P/M Composite

The d_33_ piezoelectric coefficient of the piezoelectric film was closely related to the piezoelectric properties of the material itself, and its value can reflect the strength of the piezoelectric properties of the material. The d_33_ piezoelectric coefficient of the composite film is shown in Figure 3g. It can be observed from the figure that with the increase of MXene content, the d_33_ piezoelectric coefficient of the composite film began to increase gradually, from −3.43 pC/N to −26.01 pC/N. When the content of MXene increased from 1.00% to 1.25%, the piezoelectric coefficient of d_33_ decreased from −26.01 pC/N to −19.16 pC/N. The composite film with 1.00% MXene content had the largest piezoelectric coefficient, because its piezoelectricity came from the β crystal phase of the all-trans conformation. The higher the proportion of β phase, the stronger the electrode and piezoelectricity of the material. The test results of d_33_ piezoelectric coefficient were consistent with the calculated content trend of β phase, which also verified the test results.

Figure 3h showed the piezoelectric output of PVFT/MXene composite films with different contents under 15 N stress, and the frequency was 0.5 Hz. The piezoelectric output of the pure PVFT film was 0.24 V. With the increase of MXene content, the output voltage of the composite film began to increase. The maximum output voltage was 4.62 V to 5.68 V at 1.00% MXene, and the average output voltage was above 5 V. When the content of MXene increased to 1.25%, the output voltage began to decrease, falling to 3.59 V to 4.11 V. In summary, the composite film with 1.00% MXene had the best piezoelectric output performance. Therefore, P/M-1.00 was selected to composite with PTP hydrogel. In order to evaluate the piezoelectric properties of the composite film under different stresses, the piezoelectric output effects under different stresses (5 N, 10 N and 15 N) were tested on the P/M-1.00 composite film with the best piezoelectric output performance, as shown in Figure 3i. The output voltage was 2.3–2.79 V under 5 N stress. As the stress increased, the output voltage gradually increased. At 15 N, the maximum output voltage was 4.38–5.27 V. It can be intuitively observed that the piezoelectric output of the composite film was proportional to the stress applied to the surface. In order to explore the piezoelectric output performance of the composite dressing, the piezoelectric output test of the encapsulated composite dressing was carried out using mechanical stress of 5 N, 10 N and 15 N at a frequency of 0.5 Hz, and the piezoelectric performance was judged by the open circuit voltage. As shown in Figure 3j, the open circuit voltages under three stresses were 0.02–0.03 V, 0.04–0.05 V and 0.07–0.10 V, respectively. The results showed that the open circuit voltage increases with the increase of the input mechanical stress. The almost constant output voltage after multiple measurements proved that the composite dressing has a stable piezoelectric output and does not attenuate, and the composite dressing had excellent piezoelectric properties.

The effect of doping MXene on the conductivity of the composite film was shown in Figure 3k. With the increase of MXene content, the maximum current of the composite film increased slowly and then decreased. The maximum current of different MXene content was 0.039, 0.041, 0.046, 0.059, 0.068 and 0.027 μA, respectively. The P/M-1.00 composite film exhibited the maximum current, which was the optimal conductivity. This was related to the content of MXene. The conductivity of MXene was very strong, and its conductivity can reach up to 9880 S/cm. However, the conductivity of P/M-1.25 was the worst. Although the increase of the content can enhance the conductivity of the composite film, the excessive content will also lead to the agglomeration of MXene. At the same time, it will also make the solution sticky and increase the thickness of the composite film when casting the film. This also revealed a factor affecting the poor piezoelectric properties of the P/M-1.25 composite film. The P-E loop and remanent polarization of P/M composite films with different contents were shown in Figure 3l and Figure S4. It can be seen that with the increase of MXene content, the remanent polarization (Pr) increases first and then decreases. The change trend of remanent polarization was related to the content of β crystal phase. The β crystal phase decreased from 1.00% to 1.25%, and the remanent polarization Pr also decreased from 0.98 μC/cm^2^ to 0.38 μC/cm^2^, showing the same change trend. In addition, the hysteresis loop shapes of PVFT composite films with different MXene contents were similar, indicating that the composite films had polarization reversal behavior. On the whole, P/M-1.00 composite film had the largest Pr, which was consistent with the measured piezoelectric output and piezoelectric coefficient results. This was because MXene can attract charge inside the composite film, change the arrangement of dipoles, and enhance the electrical properties of the composite film.

From Figure S5b, it can be seen that with the increase of MXene content, the surface potential on the composite film showed a trend of increasing first and then decreasing. The surface potential values were −15.0, −37.8, −57.5, −69.1, −78.4 and −61.5 mV, respectively. The change trend was basically consistent with the piezoelectric and ferroelectric tests. Among them, the P/M-1.00 composite film had the best negative surface potential, which was −78.4 mV. This was because the β phase usually had higher piezoelectric properties and it had larger electric dipole moment. In the piezoelectric film with high content of β phase, the crystal structure was more orderly and the average value of the electric dipole moment was larger. This may lead to a stronger electric field on the membrane surface, thereby increasing the surface potential. In addition, we also explored the electrical stability of the composite film by testing the surface potential within 28 days. The surface potential of the composite film was detected every 7 days until the 28th day, as shown in Figure S5a. The surface potential of all composite membranes decreased within 28 days. The surface potential of PVFT and 0.25% MXene composite membranes did not decrease significantly, while the other composite membranes decreased significantly. This may be due to the accumulation of water molecules on the surface of the composite membrane, forming a water film, thereby reducing the surface potential. Almost all the film surface potential decreased within 10%, still showing good electrical properties. Only P/M-0.50 composite membrane surface potential decreased by 22.1%. Taken together, the P/M composite membrane exhibited appropriate surface negative potential, which could stimulate cell migration and proliferation and help accelerate the wound healing process.

### 3.5. Physical and Chemical Properties of the Hydrogel

**Figure 4:**
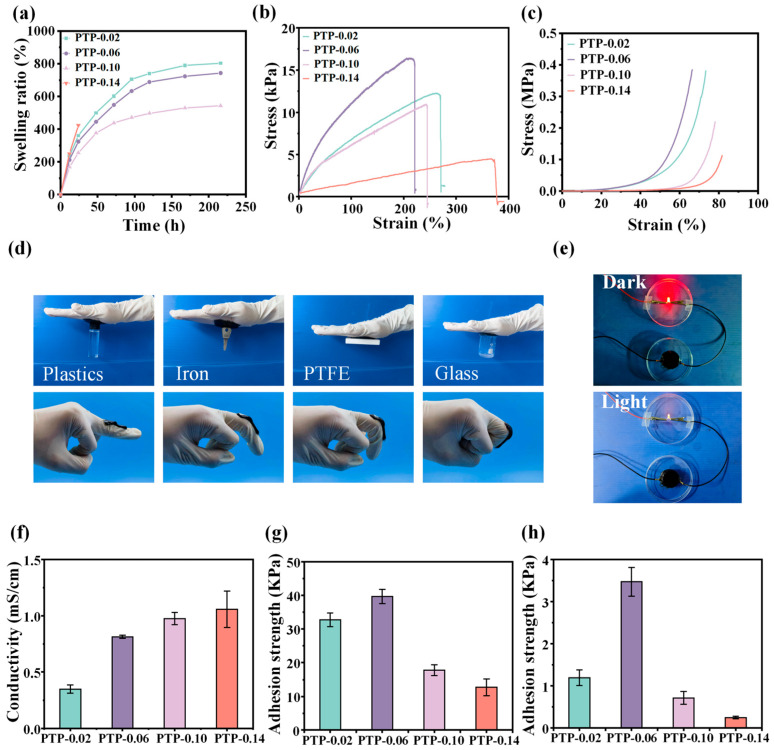
(**a**) Solubility properties, (**b**) stretch stress–strain curve, and (**c**) compression stress–strain curve of hydrogels plotted for different ANI dosages. (**d**) Adhesion diagram of hydrogel to different materials and different degrees of bending of composite dressings on the knuckles. (**e**) PTP hydrogel connects LED lights in dark and bright environments. (**f**) Conductivity of hydrogel with different contents of ANI (n = 3). Adhesion properties of hydrogels to (**g**) pig skin and (**h**) P/M film plotted for different ANI contents (n = 3).

According to the swelling performance diagram of different ANI content shown in Figure 4a, it can be seen that with the increase of ANI content, the maximum equilibrium swelling rate of the hydrogel decreased significantly, and the maximum equilibrium swelling rate decreased from 803% to 543%. This was because PANI caused the hydrogel to have denser network structure. However, when the ANI content was 0.14 mL, the hydrogel was broken in contact after swelling for 24 h. This was due to the rigid structure of the PANI molecular chain. Excessive PANI could lead to the loose structure of the hydrogel and reduce the stability. As shown in Figure 4b,c, the tensile and compressive properties of the hydrogel were significantly affected by the material concentration. When the ANI content increased from 0.02 mL to 0.14 mL, the tensile strength and compressive modulus of the corresponding hydrogel samples showed the same trend, increasing first and then decreasing. The hydrogel with the largest ANI content had the lowest tensile strength. It was worth noting that PTP-0.06 hydrogel had the maximum tensile strength and compressive modulus. This was because when the content was low, the synthesis of PANI molecular chains could help the hydrogel to form denser network structure. However, when the content continued to increase, the rigid structure of PANI could reduce the overall structural strength, which meant that too-high content will lead to competition with the cross-linked structure of PAM in cross-linking, reducing the mechanical strength. PTP hydrogel had good adhesion properties, which can effectively adhere to small glass bottles filled with water, keys (iron), polytetrafluoroethylene (PTFE) plates and small beakers. The composite dressing can meet the finger joint bending 90° without falling (Figure 4d).

Figure 4e showed a light-emitting LED lamp connected with prepared PTP-0.06 hydrogel, powered by 3 V power supply. It can be seen that the LED lamp had bright light in dark environment, indicating that the PTP-0.06 hydrogel had excellent electrical conductivity. To enhance comprehensive mechanical properties and electrical conductivity, PTP-0.06 hydrogel was selected to be compounded with P/M piezoelectric film. The conductivity change diagram of the hydrogel was shown in Figure 4f. As the content of ANI increased, the conductivity also increased, which were 0.35 mS/cm, 0.81 mS/cm, 0.98 mS/cm and 1.06 mS/cm, respectively. Because PANI was dispersed in the hydrogel and formed a conductive network, its molecular structure contained conjugated benzene rings, which formed electronic conduction channels through π-π stacking, making PANI exhibit good electrical conductivity. In addition, Fe^3+^ and APS were oxidizing substances, while PANI had reducibility. Therefore, PANI can also achieve electron doping through redox reaction to further improve the conductivity. Dressings with conductive properties can enhance the endogenous current in the skin, which led to the migration of neutrophils, macrophages and keratinocytes to the wound site, thereby accelerating wound healing. Therefore, these conductive hydrogels exhibited similar conductivity to the skin, which will be beneficial to wound healing.

As shown in Figure 4g,h, it can be seen that the adhesion of the hydrogel to the pig skin was greater than that of the film. With the increase of ANI content, the adhesion performance of the hydrogel also showed the trend of increasing first and then decreasing. This was because when the amount of ANI added was small, the crosslinking density was small, the cohesion was small, and the hydrogel was easy to move inside when adhering, resulting in a decrease in adhesion. Therefore, with the increase of ANI content, the adhesion was enhanced. However, with the continuous increase of ANI content, on the one hand, this may be due to the excessive crosslinking leading to the decrease of the number of phenolic hydroxyl groups on the surface. On the other hand, it may also be due to the excessive ANI content, which reduced the crosslinking degree of PAM and reduced the cohesion.

The rheological properties of PTP hydrogels were studied to evaluate the mechanical properties of their hydrogels. In the strain range of 0.1% to 104%, the storage modulus (elastic modulus, G′) was always higher than the loss modulus (viscous modulus, G″) until the strain reached 1755%. When the strain continued to increase, G′ showed a sharp decrease and was lower than G″, indicating that the hydrogel network collapsed and changed from the gel state to the sol state (Figure S6a). In the frequency range of 0.1–100 Hz, the G′ of the hydrogel was greater than G″, indicating that the hydrogel had good stability (Figure S6b). The results showed that the PAM-TA-PANI hydrogel had good mechanical properties, making it mechanically compatible as wound dressing.

Moreover, we systematically evaluated the degradation behavior of PT and PTP hydrogels in SBF simulated body fluid. The results showed that both hydrogels exhibited slow degradation characteristics, and the cumulative degradation rate was less than 12% within 14 days, indicating that they could maintain more than 88% structural integrity during the clinical use cycle (1–2 weeks) of acute wounds, meeting the functional requirements of the dressing during wound healing. Compared with PT hydrogel, PTP hydrogel showed a lower degradation rate (11.1% in 14 days) due to the introduction of polyaniline (PANI), a large number of hydrogen bonds and π-π stacking in its three-dimensional network, which significantly improved the network density and antioxidant stability (Figure S7). This excellent structural stability provided guarantee for its long-term antibacterial and repair treatment of infectious wounds.

### 3.6. Photothermal Property Analysis

**Figure 5:**
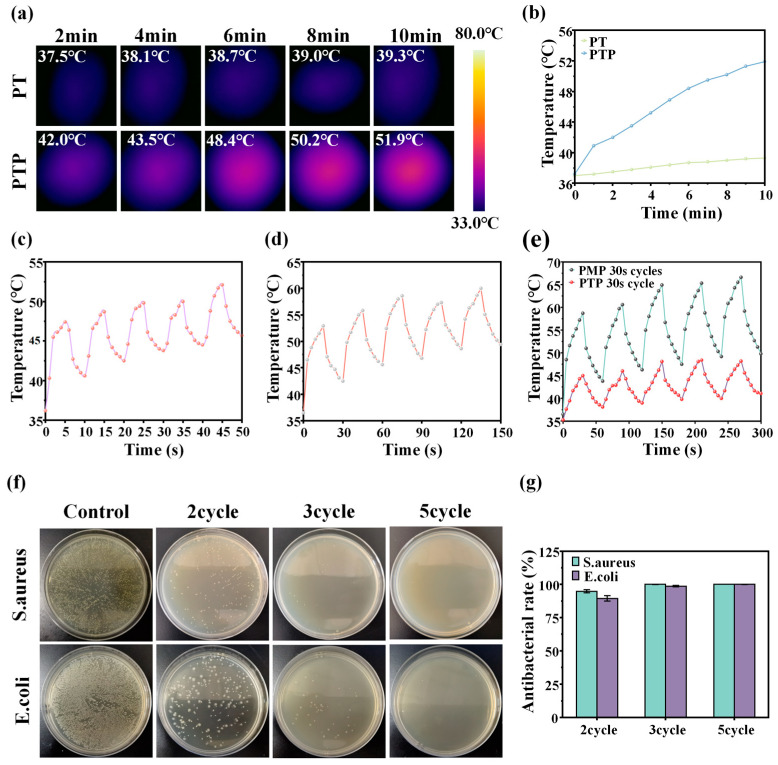
(**a**) Near-infrared thermograms of PT and PTP hydrogels in 10 min and (**b**) temperature rise curves of PT and PTP hydrogels. The temperature change diagram of the composite dressing in (**c**) 5 s and (**d**) 15 s cycles under infrared light irradiation. (**e**) The temperature change diagram of PTP hydrogel and composite dressing in 30 s cycle under infrared light irradiation. (**f**) Images of *Staphylococcus aureus* and *Escherichia coli* plate colony and (**g**) quantification of antibacterial rate (n = 3).

Biomaterials with excellent photothermal properties can absorb light energy and convert it into heat energy during wound repair, which can promote healing, inhibit infection, relieve pain and control wound exudation [33,34]. Figure 5a showed the photothermal image of different hydrogels selected within 10 min of NIR (0.1 W/cm^2^) irradiation, and Figure 5b showed the temperature rise map of different hydrogels. The PT hydrogel can be seen to increase from 38 °C to 39.3 °C. After adding PANI, the hydrogel temperature increased to 51.9 °C. This was because the phenolic hydroxyl structure in TA oxidized to form blue-black quinone substance, which slightly improved the photothermal performance. Polyaniline, as polymer material with conjugated structure, had high light absorption capacity, especially in the visible light range [35]. Therefore, the addition of TA and PANI can enhance the light absorption ability of the hydrogel and improve the photothermal conversion efficiency.

**Figure 6:**
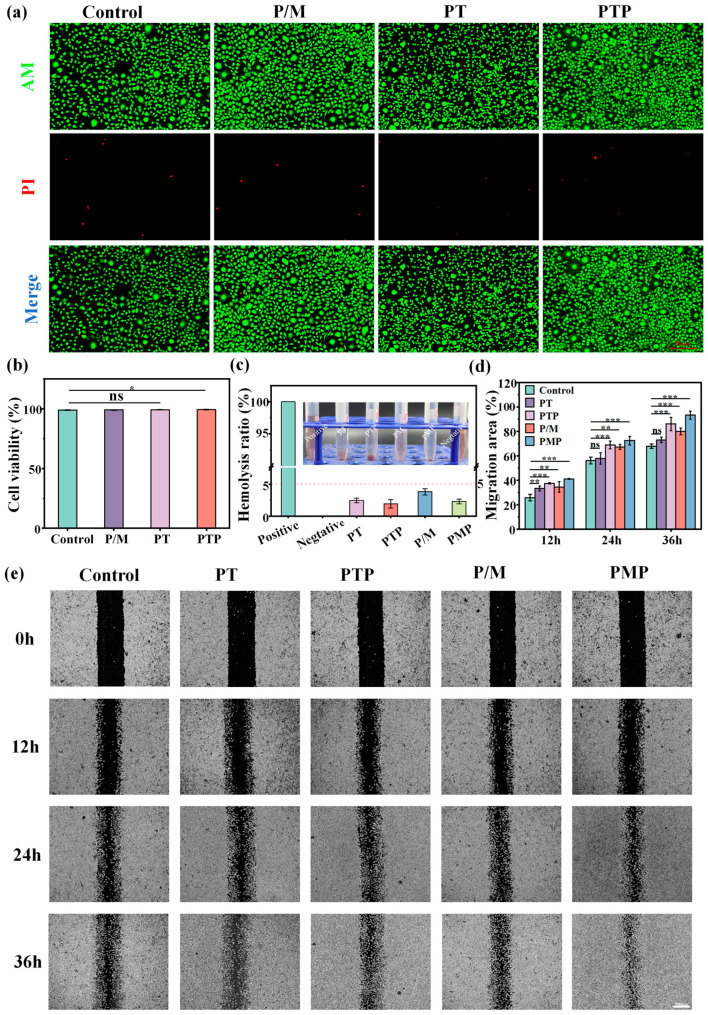
(**a**) Fluorescence images of live/dead staining. Scale bars: 50 μm. (**b**) Cellular activity of L929 cells after co-culture of PT hydrogel, PTP hydrogel and P/M piezoelectric film for 48 h (n = 3). (**c**) The blood compatibility of PT hydrogel, PTP hydrogel, P/M membrane and PMP (n = 3). (**d**) Qualitative analysis of cell migration (n = 3). (**e**) Representative images of cell migration assay of PT hydrogel, PTP hydrogel, P/M piezoelectric film and PMP dressing at different time points. Scale bars: 500 μm * *p* < 0.05, ** *p* < 0.01 and *** *p* < 0.001.

MXene has excellent photothermal properties, and compounding MXene into PVFT film may change its photothermal properties. The pre-experiment showed that when the irradiation time of the composite membrane exceeded 30 s, the softening deformation phenomenon would occur due to the sharp rise of temperature, which would affect the physical and chemical properties of the membrane. The composite membrane rapidly heated up under the cyclic NIR irradiation, and the temperature reached 87.7 °C after 5 s (Figure S8). This showed that the composite film had excellent photothermal properties, light absorption performance and good photothermal conversion efficiency after adding MXene. Therefore, it was decided to use three cycles of 5 s, 15 s and 30 s to irradiate the composite dressing with NIR. As shown in Figure 6c, the temperature of the composite dressing raised rapidly under cyclic NIR irradiation. The temperature reached 50 °C at a cycle of 5 s. When the irradiation time increased to 15 (Figure 5d) and 30 s (Figure 5e), the temperature rose to 57.3 °C and 65.3 °C, respectively. However, the temperature of PTP hydrogel only increased to 48.4 °C under 30 s NIR irradiation. This indicated that the photothermal heating capacity of the composite dressing was higher than that of PTP hydrogel, and it had good photothermal stability.

### 3.7. In Vitro Antibacterial Properties Analysis

The ideal wound dressing should have good antibacterial properties, reduce the number of pathogens in the wound, treat inflammation and promote wound healing. As shown in Figure S9a,b, the antibacterial schematics of the two hydrogels were presented. The control group was PT hydrogel, and the hydrogel showed slight antibacterial effect compared with the blank group. This was because TA can change the cell structure and destroy the cell membrane with an antibacterial effect [36]. With the addition of PANI, the inhibitory effect of the hydrogel on the two bacteria was enhanced, and the effect on *Staphylococcus aureus* was more obvious. This was because PANI had redox properties and can undergo redox reactions with microorganisms in the environment, thereby destroying the cell structure and playing an antibacterial role. Subsequently, PTP hydrogel was subjected to NIR irradiation for 10 min, and the results showed that the inhibition rate of the two bacteria reached almost 100%. This was due to the good photothermal ability of TA-Fe complex and PANI, which made the hydrogel warm up under NIR irradiation, and the cell membrane of bacteria was damaged by overheating and led to death. In order to explore the effect of different NIR irradiation time on the antibacterial effect of PTP hydrogel, the antibacterial effect of 3 min and 5 min under NIR irradiation was investigated and compared with that under 10 min irradiation, as shown in Figure S9c,d. With the increase of irradiation time, the antibacterial rate increased and the number of colonies decreased. This was because as the NIR irradiation time increased, the hydrogel gradually heated up, and the higher the temperature, the better the killing effect on bacteria. At 10 min, the maximum antibacterial rate reached 99.9%. In order to explore the effect of different NIR irradiation cycles on the antibacterial effect of the composite dressing, the antibacterial effects of two cycles and three cycles under NIR irradiation were investigated and compared with those under five cycles of irradiation, as shown in Figure 5f,g. It can be seen from the figure that when NIR was irradiated for two cycles, the antibacterial rates of the composite dressing against *Staphylococcus aureus* and *Escherichia coli* were 94.8% and 89.4%, respectively, showing excellent antibacterial properties. When NIR was irradiated for three cycles, the antibacterial rates were 99.9% and 98.6%, respectively. The composite dressing had excellent photothermal conversion performance. The longer the irradiation time and the higher the temperature, the better the antibacterial effect. When the NIR irradiation time was increased to five cycles, the antibacterial rate against the two bacteria reached 100%, indicating that the prepared composite dressing could achieve an efficient antibacterial effect under NIR irradiation.

### 3.8. In Vitro Cytocompatibility of the Composite Dressing

Good biocompatibility is essential for the clinical application of wound dressings. Figure 6a showed the live and dead cell fluorescence staining image of L929 cells co-cultured with the prepared P/M composite film, PT and PTP hydrogel materials for 48 h. The results showed that during the 48 h co-culture period, the composite membrane and the two hydrogels had less damage to the cells, and the cell survival rate reached more than 98%, showing good cell compatibility. Figure 6b demonstrated that the cell viability was good, which indicated that the prepared composite membrane, PT and PTP hydrogels and composite dressings as biological materials were completely non-toxic to cells and had good biocompatibility. This was consistent with the above results. It can be seen from Figure 6c that the hemolysis rate of all materials was less than 5%, which proved that they had excellent blood compatibility as wound dressings.

### 3.9. Cell Migration Assessment

The migration of L929 cells cultured on the hydrogel was evaluated by scratch assay. The cell migration was recorded at 0, 12, 24 and 36 h (Figure 6d,e). It can be seen that the cell migration rate of PT hydrogel and P/M was slightly higher than that of the blank group after 36 h, which was 73.02% and 79.92%, respectively. The cell migration rate of PTP group and PMP group was significantly higher than that of other groups, especially the PMP group, which was as high as 93.27%. Therefore, PTP conductive hydrogel and P/M can synergistically promote cell migration. It can be a potential candidate for wound dressing applications.

### 3.10. Analysis of the Internal Infection Wound Healing by the Composite Dressing

**Figure 7:**
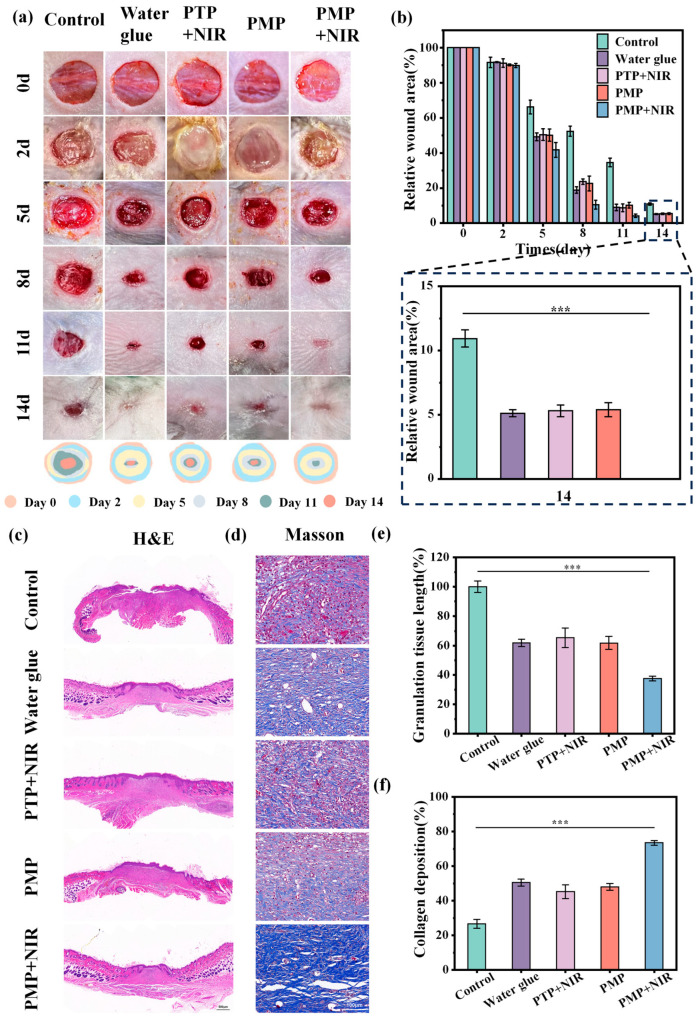
(**a**) Representative photographs of infected wounds at different time points during the treatments and schematic images of wound closure trace during 14 days. (**b**) Relative wound closure area of the mice from day 0 to day 14 after different interventions (n = 6). (**c**) Representative H&E staining of the wound beds on day 14. Scale bars: 500 μm. (**d**) Representative Masson trichrome staining photographs of the wound sites in each group. Scale bars: 100 μm. (**e**) Quantitative analysis of granulation tissue width on day 14 (n = 6). (**f**) Quantitative analysis of collagen deposition estimated from images of Masson trichrome staining (n = 6). *** *p* < 0.001.

In order to evaluate the wound healing effect of the composite dressing, a wound defect model infected with *Staphylococcus aureus* was established on the backs of mice. The wounds were divided into five groups: blank group, commercial medical dressing water glue group, PTP+NIR group, PMP group and PMP+NIR group. The blank group did not receive any treatment, the PTP+NIR group was irradiated with NIR (1 W/cm^2^) for 10 min on the 1st day after hydrogel treatment, and the PMP+NIR group was irradiated with NIR (1 W/cm^2^) for 30 s for five cycles. (All composite dressing groups deform the composite membrane through the mouse’s own activities and joint bending to produce a piezoelectric effect). Figure 7a,b showed the results of wound repair at 0, 2, 5, 8, 11 and 14 days after dressing treatment. It can be seen that the infected wound area of all wounds decreased on the 5th day, and the wound repair effect of commercial medical dressing water glue and PMP+NIR group was the best, indicating that electrical stimulation and photothermal therapy played a synergistic role in wound treatment. On the 11th day, the PTP+NIR group, the PMP group and the PMP+NIR group all had different degrees of repair effect on the wound. The PTP+NIR and PMP groups had positive effect on wound healing, and their healing effects were better than the blank group. Among them, the PMP+NIR group showed better therapeutic effect than the commercial medical dressing water glue, and the wound closure rate was significantly higher than other groups. This proved that the composite dressing can achieve faster wound closure through electrical stimulation and photothermal therapy, and can provide electrical stimulation and optimal healing conditions for the wound. Due to the current experimental timeframe of 14 days, the long-term dynamics beyond this period were not captured. Future studies extending the observation period to 4–8 weeks are needed to assess the sustained effects of the observed phenomena.

To further analyze epidermal regeneration and wound repair, H&E staining was performed on the granulation tissue on the wound bed. According to the results of wound closure rate, the wound treated with PMP+NIR irradiation had narrower granulation tissue width and thicker regenerated epithelial tissue than other wounds, indicating that the piezoelectric composite dressing with electrical stimulation and photothermal antibacterial function can improve the wound healing microenvironment (Figure 7c,e). In order to study the biological mechanism of the wound repair process, Masson trichrome staining was performed to evaluate the distribution of collagen in the wound site. As an indispensable part of the skin, the synthesis, deposition and orientation of collagen played a crucial role in wound repair. The presence of collagen deposition was conducive to the healing process. Obviously, the PMP+NIR group had more deposition and highly oriented collagen structure, showing better extracellular matrix remodeling and epithelial tissue reconstruction (Figure 7d,f).

## 4. Conclusions

The core scientific novelties of our research are as follows: Firstly, we constructed a composite dressing of MXene-doped PVFT piezoelectric film and self-adhesive high-conductivity hydrogel, which solved the core pain points of existing dressings, including poor wound interface adhesion, low electric field transmission efficiency, and incompatibility with moist wound microenvironment. Secondly, through MXene interface engineering, we increased the β phase content of PVFT from 54% to 85.6%, the piezoelectric coefficient d_33_ from −3.43 pC/N to −26.01 pC/N, and the output voltage up to 5.68 V. The dressing can realize self-driven endogenous electrical stimulation driven by daily human physiological activities, which was difficult to achieve with most previous low-output piezoelectric dressings. Thirdly, we systematically confirmed the “1 + 1 > 2” synergistic effect between photothermal antibacterial and endogenous electrical stimulation through controlled experiments and statistical analysis. Benefiting from the synergistic effect, we achieved 100% sterilization with only 1/4 of the traditional NIR irradiation dose, breaking the trade-off between antibacterial efficiency and biosafety, and significantly accelerated wound healing. Finally, the dressing has excellent skin adhesion, mechanical properties highly matched with human skin, simple preparation process, and flexibly adjustable therapeutic parameters, which greatly improved the clinical translation potential compared with previous similar dressings.

In summary, we developed a novel kind of piezoelectric composite dressing with photothermal antibacterial function and endogenous electrical stimulation for infected wound healing. The piezoelectric properties, photothermal properties and antibacterial properties of the composite dressing were investigated. The wound healing effect of the composite dressing was explored by the infected wound mice model. The results showed that the composite dressing had excellent piezoelectric output effect, and can stably output a voltage of 0.07–0.10 V under the stress of 15 N. The excellent photothermal effect of the composite film made the temperature of the composite dressing reach 65.3 °C under 30 s cycle NIR irradiation, which was much higher than the heating rate of the hydrogel. Therefore, when the same photothermal antibacterial effect was achieved, the NIR irradiation time required for the composite dressing was also shorter. In the wound healing experiment, the composite PMP+NIR group showed the best healing effect, which provided new insight for the design of new composite piezoelectric biomaterials and had great application prospects in wound treatment.

## Figures and Tables

**Table 1:** Preparation parameters of P/M composite membrane.

Abbreviation	PVFT/g	DMF/mL	MXene Dispersion /mL
PVFT	1	7.33	0
P/M-0.25	1	6.83	0.5
P/M-0.50	1	6.33	1.0
P/M-0.75	1	5.83	1.5
P/M-1.00	1	5.33	2.0
P/M-1.25	1	4.83	2.5

## Data Availability

The original contributions presented in this study are included in the article. Further inquiries can be directed to the corresponding authors.

## Supplementary Materials

The following supporting information can be downloaded at: https://www.mdpi.com/article/10.3390/pharmaceutics18050607/s1.
