# Supplementary material for: Photothermally Antibacterial Piezoelectric Composite Dressing Synergized with Endogenous Electrical Stimulation for Wound Healing
Source: Pharmaceutics. 2026 May 15;18(5):607. doi: 10.3390/pharmaceutics18050607 (PMC13210367; doi:10.3390/pharmaceutics18050607)
Supplement: Supplementary file 1 [file pharmaceutics-18-00607-s001.zip › pharmaceutics-4238333-supplementary.pdf]

Supplementary Information

Photothermally Antibacterial Piezoelectric Composite Dressing Synergized with Endogenous Electrical Stimulation for Wound Healing

Hao-Zhe Yu [1,†], Guan-Yong Deng [1], Nan Gao [2,†], Li-Hong Fan [1], Jian-Wen Wang [2], Xing-Jian Liu [1], Wei Zhang [1], Shi-Lin Tian [1], Yu-Xiong Weng [2], He-Shuang Dai [1], Yi-Wen Zhang [1] and Huan Deng [1,*]

[1] School of Chemistry, Chemical Engineering and Life Sciences, Wuhan University of Technology,
Wuhan 430070, China; 346355@whut.edu.cn (H.-Z.Y.); 349065@whut.edu.cn (G.-Y.D.);
lhfan@whut.edu.cn (L.-H.F.); 349070@whut.edu.cn (X.-J.L.);
346372@whut.edu.cn (W.Z.); 346344@whut.edu.cn (S.-L.T.);
daiheshuang@whut.edu.cn (H.-S.D.); 374851@whut.edu.cn (Y.-W.Z.)

[2] Department of Hand Surgery, Union Hospital, Tongji Medical College, Huazhong University of Science and Technology, Wuhan 430022, China;
gaonan1112@gmail.com (N.G.); wjw19980315@163.com (J.-W.W.);
yxweng1218@163.com (Y.-X.W.)

* Correspondence: denghuan@whut.edu.cn; Tel.: +86-15907145126

† These authors contributed equally to this work.

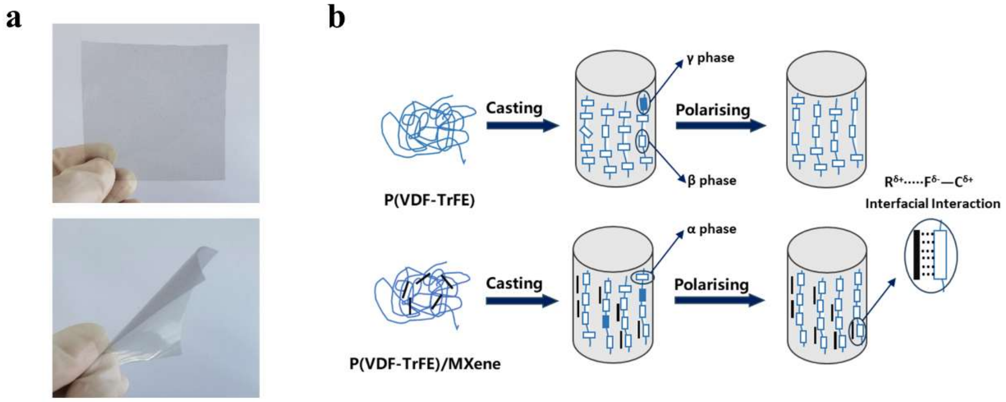

Figure S1. (a) Front view (left) and side view (right) of a P/M composite film. (b) β-phase content of PVFT composite membranes with different concentrations of MXene.

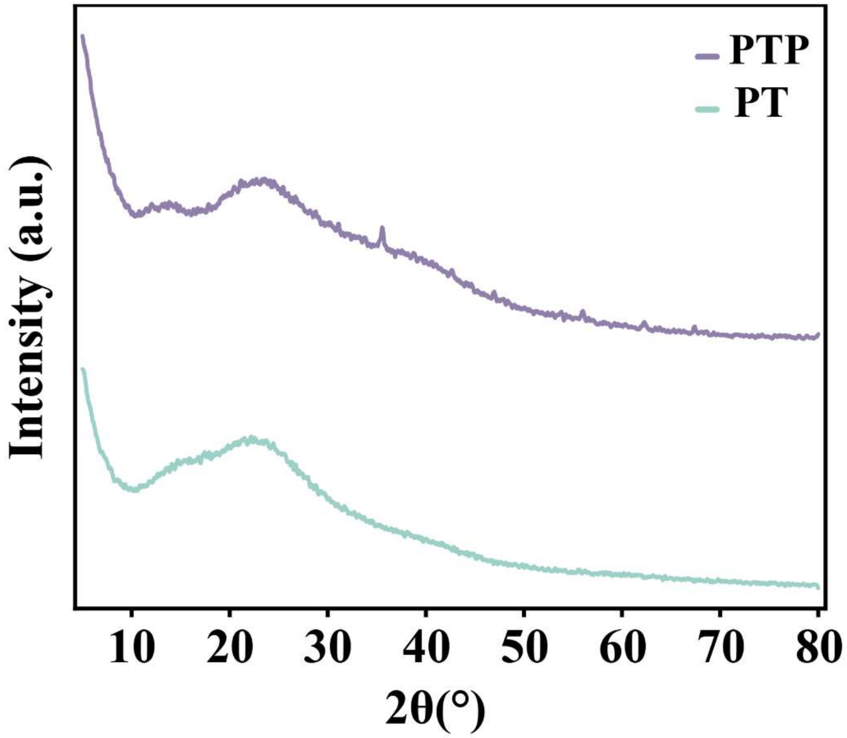

Figure S2. The XRD patterns of PT and PTP hydrogels.

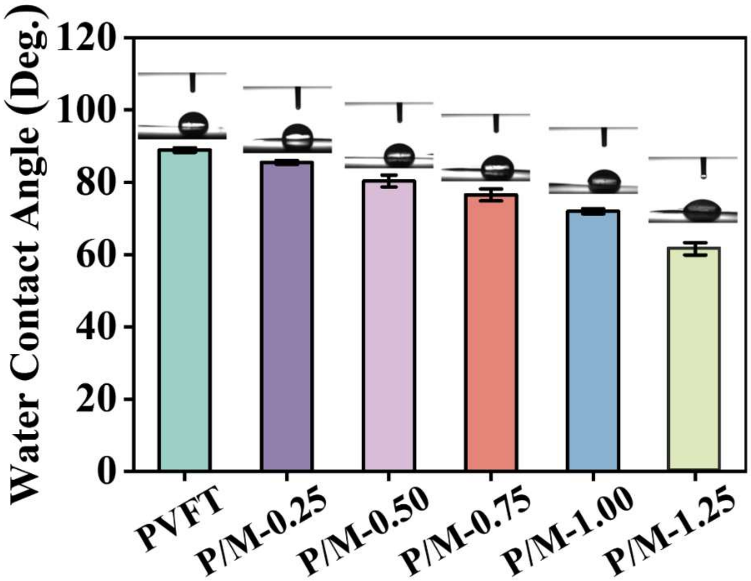

Figure S3. The surface water contact angle changes of the composite membranes with different MXene contents (n = 3).

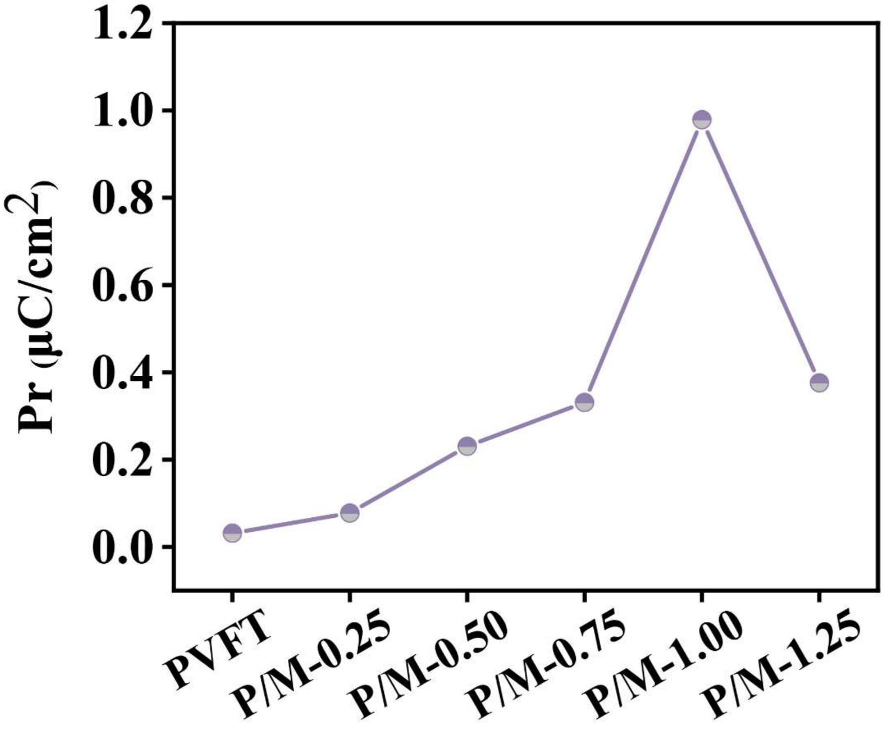

Figure S4. Residual polarization of P/M composite membranes with different contents.

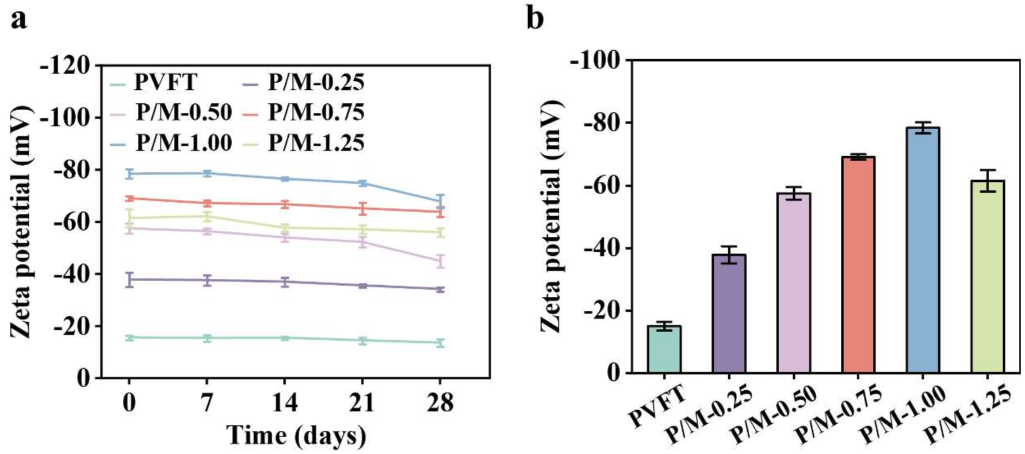

Figure S5. (a) Surface potential and (b) surface potential stability of P/M composite membranes with different contents (n = 3).

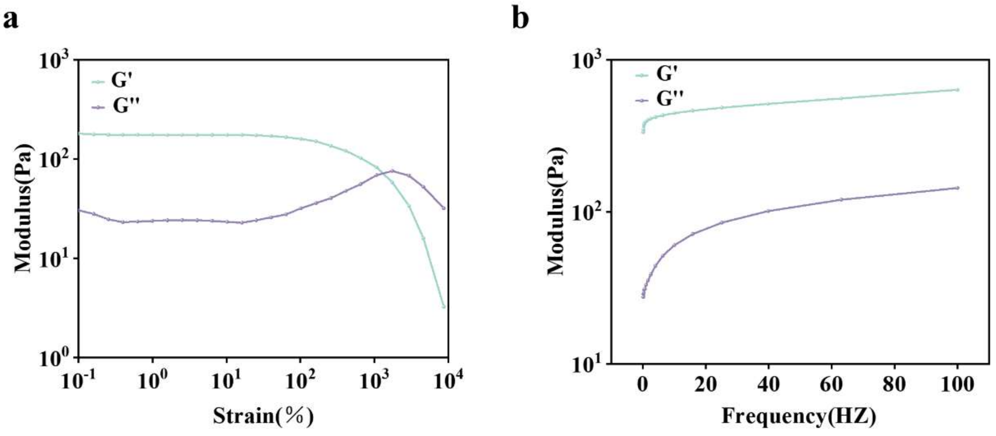

Figure S6. Characterization of the rheological properties of PAM-TA-PANI hydrogels. (a) Frequency sweep measurement of the hydrogel. (b) Strain sweep measurement of the hydrogel.

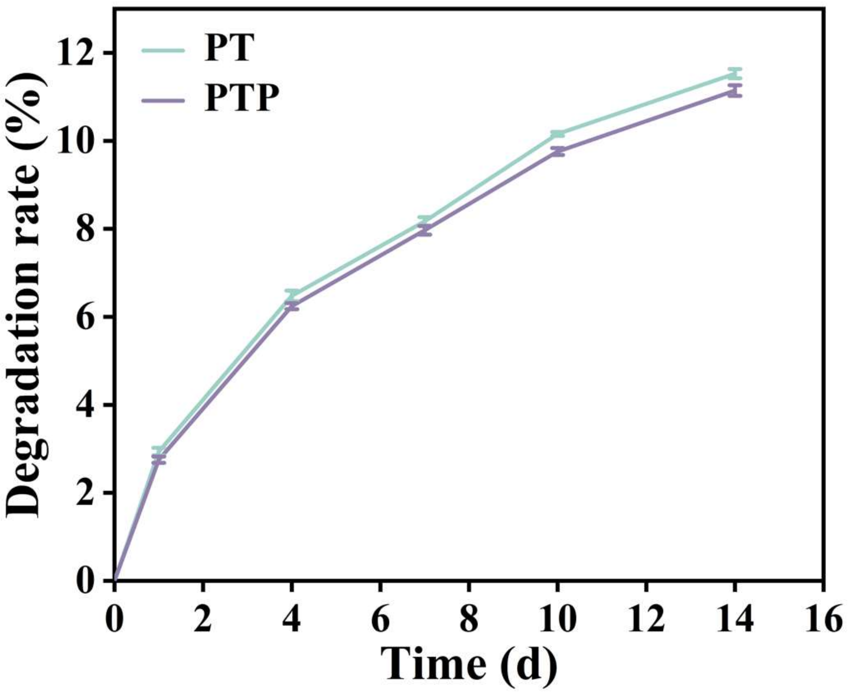

Figure S7. The degradation rate of PT and PTP hydrogels in SBF simulated body fluid within 14 days (n = 3).

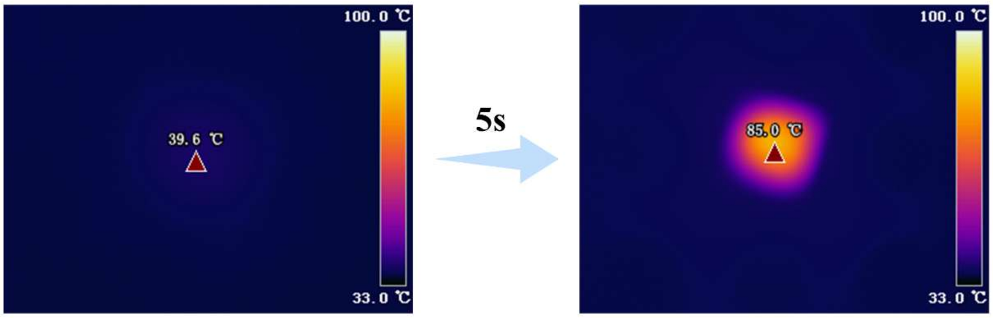

Figure S8. The near-infrared heating map of PVFT/MXene composite film irradiated from (a) 0s to (b) 5s at 1 W/cm^2 was shown.

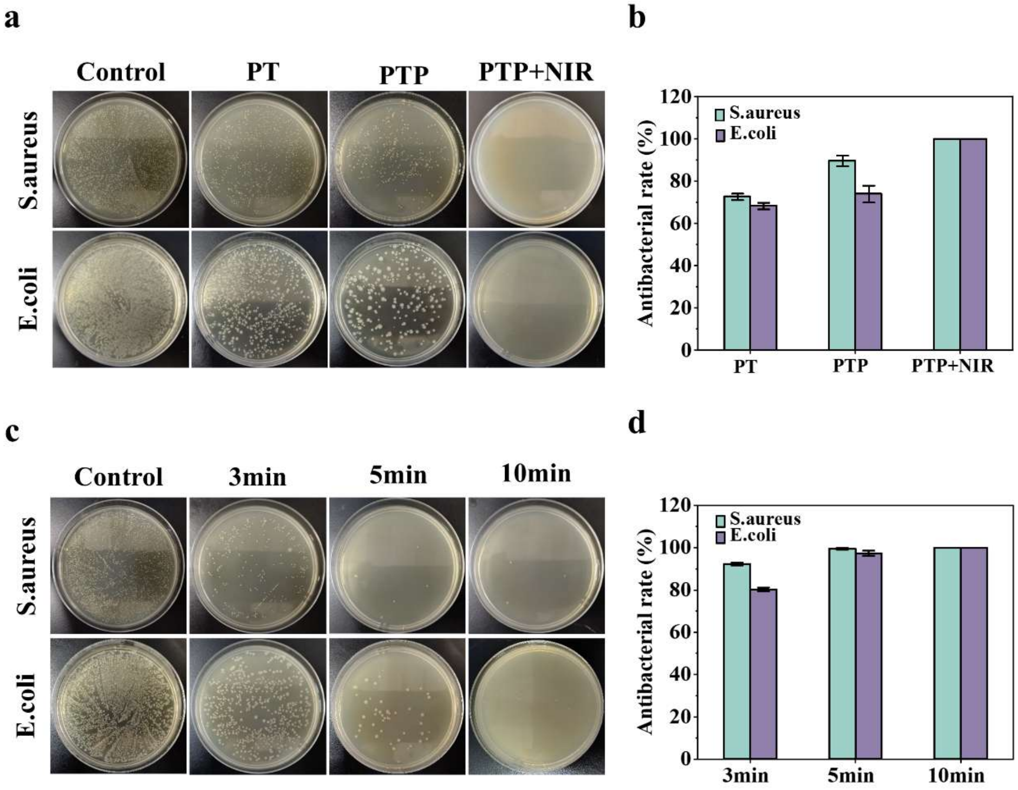

Figure S9. (a) Plate colony images and (b) antimicrobial rate of Staphylococcus aureus and Escherichia coli on PAM-TA and PAM-TA-PANI hydrogels (n = 3). (c) Images of Staphylococcus aureus and Escherichia coli colonization plate and (d) antimicrobial rate of PAM-TA-PANI hydrogels with different NIR irradiation time points (n = 3).
